# Association of the End-Stage Renal Disease Treatment Choices Payment Model With Home Dialysis Use at Kidney Failure Onset From 2016 to 2022

**DOI:** 10.1001/jamanetworkopen.2023.0806

**Published:** 2023-02-27

**Authors:** Kirsten L. Johansen, Shuling Li, Jiannong Liu, Eric D. Weinhandl, David T. Gilbertson, Iris Kou, Christopher Knapp, James B. Wetmore

**Affiliations:** 1 Chronic Disease Research Group, Hennepin Healthcare Research Institute, Minneapolis, Minnesota; 2 Department of Nephrology, University of Minnesota, Minneapolis; 3 Satellite Healthcare, Crestwood, Illinois

## Abstract

**Importance:**

The Centers for Medicare & Medicaid Services designed a mandatory payment model to incentivize home dialysis use: the End-Stage Renal Disease Treatment Choices (ETC). Outpatient dialysis facilities and health care professionals providing nephrology services were randomly assigned to ETC participation at the hospital referral region level.

**Objective:**

To assess the association between ETC and home dialysis use in the incident dialysis population in its first 18 months of implementation.

**Design, Setting, and Participants:**

A cohort study with controlled, interrupted time series analysis of the US End-Stage Renal Disease Quality Reporting System database was conducted, using generalized estimating equations. All adults initiating home-based dialysis in the US between January 1, 2016, and June 30, 2022, without a prior kidney transplant were included in the analysis.

**Exposures:**

Prior to vs after ETC onset in January 1, 2021, and random assignment to ETC participation of facilities and health care professionals involved in patient care.

**Main Outcomes and Measures:**

Percentage of patients started on incident home dialysis and yearly change in percentage initiating home dialysis.

**Results:**

A total of 817 177 adults initiated home dialysis during the study period, of whom 750 314 were included in the study cohort. The cohort included 41.4% women; 26.2% of the patients were Black, 17.4% were Hispanic, and 49.1% were White. Approximately half (49.6%) of the patients were aged at least 65 years. A total of 31.2% received care from health care professionals assigned to ETC participation, and 33.6% had Medicare fee-for-service coverage. Overall, home dialysis use increased from 10.0% in January 2016 to 17.4% in June 2022. Home dialysis use increased more in ETC markets than in non-ETC markets after January 2021 (by 1.07%; 95% CI, 0.16%-1.97%). The rate of increase in home dialysis use in the entire cohort nearly doubled after January 2021 to 1.66% per year (95% CI, 1.14%-2.19%) compared with before 2021, when the rate was 0.86% per year (95% CI, 0.75%-0.97%), but the difference in rate of increase in home dialysis use was not significant between ETC and non-ETC markets.

**Conclusions and Relevance:**

This study noted that, although the overall rate of dialysis use at home was greater after ETC implementation, the increase occurred more among patients in ETC markets than among those in non-ETC markets. These findings suggest that federal policy and financial incentives affected care for the entire incident dialysis population in the US.

## Key Points

**Question:**

Was initiation of the End-Stage Renal Disease Treatment Choices (ETC) payment model associated with changes in the incident dialysis population in its first 18 months of implementation?

**Findings:**

In this cohort study of 750 314 adults with chronic kidney disease, increased use of home dialysis after ETC implementation was noted among incident dialysis patients use in markets randomly assigned to ETC compared with markets that were not. Also, the overall rate of increase in the percentage of dialysis patients starting home dialysis nearly doubled.

**Meaning:**

The findings of this study suggest that financial incentives were associated with increasing home dialysis use beyond established secular trends, but the magnitude the magnitude of increase in home dialysis after ETC implementation will not achieve ambitious targets.

## Teaser

This cohort study examines changes in the use of home dialysis among patients with kidney failure after implementation of the End-Stage Renal Disease Treatment Choices payment model.

## Introduction

Dialysis performed at home can offer a sense of control, independence, self-efficacy, and flexibility, factors that are important to many patients who require maintenance dialysis.^1,2^ Use of home dialysis has been increasing over the past decade. Nevertheless, only 13% of patients undergoing dialysis in the US do so at home.^3^ The Advancing American Kidney Health Executive Order, signed by the US president in 2019, set forth ambitious targets for increased use of home dialysis in the US. Subsequently, the Centers for Medicare & Medicaid Services (CMS) introduced payment models to incentivize home dialysis, including the End-Stage Renal Disease Treatment Choices (ETC) model, in which outpatient dialysis facilities and health care professionals providing nephrology services were randomly assigned to mandatory participation at the hospital referral region (HRR) level.^4^ The HRRs assigned to ETC were announced in September 2020, and ETC was launched in January 2021. For the first 2 years, clinicians and organizations (hereafter, providers) assigned to ETC received financial incentives to transition patients with chronic kidney disease stage 4 or 5 to home dialysis rather than in-center hemodialysis (Home Dialysis Payment Adjustment).^5,6^ Beginning in July 2022, these providers are subject to a performance pay adjustment that rewards higher rates of home dialysis use, waitlisting for kidney transplant, and preemptive kidney transplant, and penalizes lower rates using national quartiles as benchmarks.

Although ETC applies only to Medicare fee-for-service (FFS) beneficiaries, it has the potential to affect all patients starting treatment for kidney failure. Clinicians may implement changes in practice, such as improved dialysis modality education, care coordination, or expanded access to peritoneal catheter placement, that are not restricted to patients with FFS coverage. To assess the hypothesis that ETC is associated with the care of all patients starting dialysis in its first 18 months, we examined changes in the use of home dialysis among incident dialysis patients in the US from January 1, 2016, to June 30, 2022, overall and according to HRR ETC assignment.

## Methods

### Cohort Selection

In this cohort study, we used data from the US Renal Data System to identify adults (age ≥18 years) who initiated dialysis treatment between January 1, 2016, and June 30, 2022, based on information from the End Stage Renal Disease (ESRD) Medical Evidence Report (Form CMS 2728). We excluded patients with a prior kidney transplant or missing dialysis facility zip code. We further excluded patients being institutionalized or receiving dialysis in a skilled nursing or long-term care facility as the primary dialysis setting. The study was reviewed by the Advarra Institutional Review Board and was exempt from review and the requirement for informed consent according to 45 CFR 46.104. This report follows the Strengthening the Reporting of Observational Studies in Epidemiology (STROBE) reporting guideline for observational studies.

The ETC payment model was proposed in July 2019, but the federal government did not release further information about the model until finalizing its specifications in September 2020, at which time participating markets were announced. Health care was disrupted by the COVID-19 pandemic in 2020. Implementation of ETC was thus delayed, and there was a transient but substantial decrease in all incident patients with kidney failure.^7,8^ Therefore, in sensitivity analyses, we also excluded patients initiating dialysis in 2020 to ensure that previously observed changes in home dialysis use that occurred in 2020^7^ would not be a factor in the pre-ETC trajectory of home dialysis uptake.

### Statistical Analysis

We examined the likelihood of patients starting dialysis in the home setting during each month from January 1, 2016, to June 30, 2022, according to HRR ETC assignment, which was obtained from the CMS database.^4^ We used a controlled interrupted time series approach. We examined change in home dialysis use over the entire study period and then divided the period into before (January 1, 2016, to December 31, 2020) and after (January 1, 2021, to June 30, 2022) implementation of ETC. Specifically, we performed a generalized estimating equation model in which we modeled the likelihood of home dialysis initiation with a binomial distribution, identity link, and an exchangeable correlation structure for patients in the same HRR. We modeled calendar month as a continuous variable to estimate the secular trend and included Fourier terms (pairs of sine and cosine functions) to address observed seasonal variation in home dialysis use.

We examined differences in home dialysis initiation between ETC and non-ETC markets before and after January 1, 2021, and whether the difference between ETC and non-ETC markets changed (by adding a term for the interaction between pre-post implementation of ETC and ETC assignment to the model). We then included the 3-way interaction of time, ETC assignment, and pre-post implementation in the initial model to examine whether any changes in use trends after ETC onset differed by ETC assignment. We sequentially removed nonsignificant interaction terms at a significance level of .05. The final model included the interaction of ETC assignment and pre-post implementation and the interaction of time and pre-post implementation.

Although our primary hypothesis was that implementation of ETC was associated with an increase in home dialysis initiation in the entire incident dialysis population, we also examined whether the association varied by Medicare FFS coverage. We repeated our modeling strategy including 2-, 3-, and 4-way interactions between Medicare FFS coverage and time, ETC assignment and pre- or post-January 2021, and then removing statistically nonsignificant interaction terms. Significant interaction terms included FFS × time × pre-post January 2021, FFS × pre-post January 2021, time × pre-post January 2021, and ETC assignment × pre-post January 2021. Stratified analyses by Medicare FFS coverage status was then performed by fitting separate models.

In a sensitivity analyses, we performed the same modeling strategy excluding 2020 data from the models and using September 2020 as the ETC starting point to capture anticipatory outcomes. Our primary analysis did not include covariates, but in an additional secondary analysis, we adjusted for age (18-44, 45-64, 65-74, and ≥75 years), race and ethnicity (Asian, Black, Hispanic, non-Hispanic White, or other [Native American and Alaskan Native, Hawaiian, and Other Pacific Islander, other/multiracial, and unknown]), insurance type (Medicare FFS or other), and rural or urban residence (urban, large rural city/town, small rural town, and isolated small rural town) using data from Form CMS 2728. We adjusted for race and ethnicity and because of known differences in the use of home dialysis across race and ethnicity groups.

We used SAS, version 9.4 (SAS Institute Inc) for all analyses. Results were considered statistically significant if 2-sided *P* values were <.05.

## Results

### Overall

**Figure 1.**
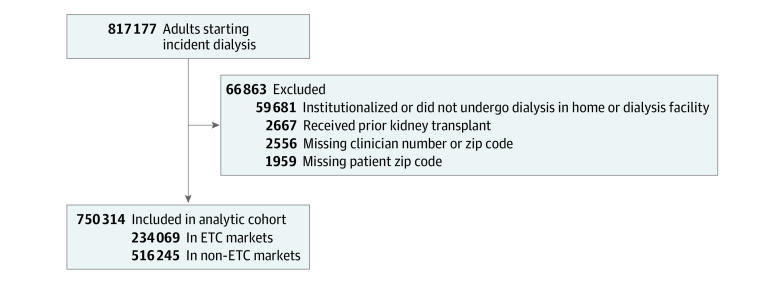
Study Flow Diagram ETC indicates End-Stage Renal Disease Treatment Choices payment model.

In total, 817 177 adults initiated dialysis for kidney failure in the US during the study period. After excluding those with prior kidney transplant (n = 2667), missing dialysis provider number or zip code (n = 2556), residing in or receiving dialysis in a long-term care facility (n = 59 681), or missing patient zip code (n = 1959), 750 314 individuals remained in the cohort (Figure 1). Of these, 41.4% were women, 26.2% were Black, 17.4% were Hispanic, and 49.1% were White. Nearly half (49.6%) of the patients were aged 65 years or older. Patient characteristics are listed in Table 1 by HRR ETC assignment. Overall, 59.5% of the patients had Medicare coverage, but only 33.6% were FFS beneficiaries with Medicare as the primary payer. As expected, based on allocation of HRRs, 31.2% of patients received care in HRRs assigned to ETC participation. Patients receiving care in ETC and non-ETC markets were similar in terms of age, sex, rural or urban residence, and insurance coverage, but the distribution of race and ethnicity differed. A higher percentage of patients starting dialysis in ETC markets was Black and a lower percentage was Hispanic, compared with patients in non-ETC markets.

**Table 1.** Patient Characteristics by Provider ETC Assignment

Variable	No. (%)	
	ETC	Non-ETC
Total	234 069	516 245
Dialysis setting		
Home dialysis	30 030 (12.8)	69 896 (13.5)
Dialysis facility/center	204 039 (87.2)	446 349 (86.5)
Age group, y		
18-44	29 230 (12.5)	62 625 (12.1)
45-64	89 497 (38.2)	196 746 (38.1)
65-74	63 581 (27.2)	139 058 (26.9)
≥75	51 761 (22.1)	117 816 (22.8)
Sex		
Female	96 859 (41.4)	213 445 (41.4)
Male	137 210 (58.6)	302 800 (58.7)
Race and ethnicity		
Asian	8697 (3.7)	27 243 (5.3)
Black	68 632 (29.3)	128 102 (24.8)
Hispanic	28 686 (12.3)	101 614 (19.7)
White	121 184 (51.8)	247 129 (47.9)
Other^a^	6870 (2.9)	12 157 (2.4)
Rural/urban status		
Urban	195 047 (83.3)	439 833 (85.2)
Large rural city/town	22 163 (9.5)	42 212 (8.2)
Small rural town	10 802 (4.6)	21 530 (4.2)
Isolated small rural town	6057 (2.6)	12 670 (2.5)
Medicare FFS insurance	79 718 (34.1)	172 245 (33.4)
Year of incident dialysis		
2016	35 255 (15.1)	77 100 (14.9)
2017	34 633 (14.8)	76 895 (14.9)
2018	35 726 (15.3)	79 251 (15.4)
2019	36 738 (15.7)	81 790 (15.8)
2020	36 304 (15.5)	79 826 (15.5)
2021	37 386 (16.0)	82 627 (16.0)
2022, Q1Q2	18 027 (7.7)	38 756 (7.5)

Abbreviations: ETC, End-Stage Renal Disease Treatment Choices; Q1Q2, quarters 1 and 2.

^a^ Other race and ethnicity includes Native American and Alaskan Native, Hawaiian, and Other Pacific Islander, other/multiracial, and unknown.

**Figure 2.**
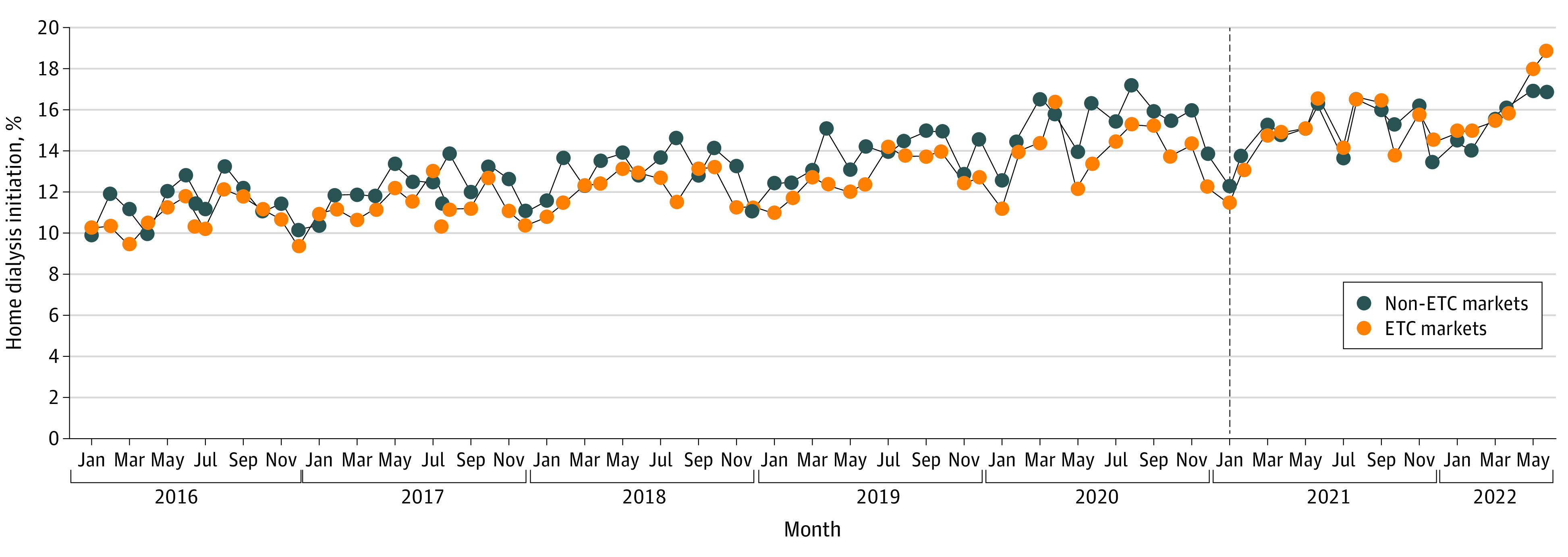
Monthly Proportion of Patients Starting Incident Dialysis Receiving Home Dialysis by End-Stage Renal Disease Treatment Choices (ETC) Assignment of Facilities and Health Care Professionals Providing Nephrology Services The black vertical line indicates the start of ETC implementation.

The percentage of patients initiating dialysis in the home setting during the entire study period (before and after ETC implementation) is shown in Figure 2. Over the course of the study period, home dialysis initiation increased from 10.0% in January 2016 to 17.4% in June 2022. Before the start of ETC (January 2016 to December 2020), the percentage of patients treated with home dialysis in ETC markets was slightly lower than in non-ETC markets (−0.75%; 95% CI, −1.96% to 0.46%)—a difference that was not statistically significant (Table 2). Overall (in the entire incident dialysis population), treatment with home dialysis increased by 0.86% (95% CI, 0.75%-0.97%) per year during the period before ETC implementation, and this slope was similar among patients in ETC and non-ETC markets (difference, −0.16% per year; 95% CI, −0.37% to 0.06%).

**Table 2.** Estimated ETC Association With the Proportion of Home Dialysis Use Using a Controlled Interrupted Time Series Analysis

Metric	Estimate (95% CI)		
	Before January 2021	After January 2021	Difference, after vs before January 2021
Use of home dialysis, %			
Difference, ETC vs non-ETC^a^	−0.75 (−1.96 to 0.46)	0.32 (−1.08 to 1.72)	1.07 (0.16 to 1.97)
Rate of increase of home dialysis use, % per year			
Overall^a^	0.86 (0.75 to 0.97)	1.66 (1.15 to 2.18)	0.81 (0.28 to 1.33)
Difference, ETC vs non-ETC^b^	−0.16 (−0.37 to 0.06)	0.85 (0.00 to 1.69)	1.00 (−0.27 to 2.27)

Abbreviation: ETC, End-Stage Renal Disease Treatment Choices.

^a^ Estimates were based on the final model with main effects of time, ETC, and pre-post implementation, and 2-way interactions of time and pre-post implementation and of ETC and pre-post implementation. The 2-way interaction of time and ETC assignment and the 3-way interaction of time, ETC assignment, and pre-post implementation were not statistically significant and thus were removed from the final model.

^b^ Estimates were based on the initial model including the 3-way interaction of time, ETC assignment, and pre-post implementation and all 2-way interactions (time and ETC, time and pre-post implementation, ETC and pre-post implementation) to examine whether any changes in use trends after ETC onset differed by ETC assignment.

After ETC onset, the percentage of patients initiating home dialysis in ETC markets was slightly higher than in non-ETC markets (0.32%; 95% CI, −1.08% to 1.72%). Although this difference between ETC and non-ETC markets was not statistically significant, the difference in the change that occurred after January 2021 was a statistically significant increase in ETC markets after ETC onset compared with non-ETC markets (1.07%; 95% CI, 0.16%-1.97%). In addition, the overall rate of increase in home dialysis use nearly doubled after ETC onset to 1.66% (95% CI, 1.15%-2.18%). There was a nominally higher rate of increase in home dialysis use in ETC markets compared with non-ETC markets after January 2021 (0.85% per year; 95% CI, 0.00%-1.69% per year), but this difference was not statistically significant. The change in the slope from before to after January 2021 also did not differ significantly in ETC compared with non-ETC markets, and this interaction term was removed from the final model.

### Associations According to Medicare FFS Coverage

Use of home dialysis was consistently lower among Medicare FFS beneficiaries than among non-FFS beneficiaries (eFigure in Supplement 1). Differences in home dialysis use between patients in ETC markets and those in non-ETC markets were similar among those with and without Medicare FFS coverage before and after ETC implementation (Table 3). Home dialysis initiation was increasing at a similar rate among patients with and without Medicare FFS coverage before ETC implementation (0.82% per year; 95% CI, 0.70%-0.94% per year for those with FFS coverage vs 0.88% per year; 95% CI, 0.74%-1.02% per year for those without). There was no significant change in the rate of increase in home dialysis initiation among Medicare FFS beneficiaries after January 2021 (0.22% per year, 95% CI, −0.45% to 0.88% per year), whereas the rate increased significantly among non-Medicare FFS beneficiaries by 1.34% per year (95% CI, 0.68%-2.00% per year), and the interaction between Medicare FFS coverage and the change in slope of increase in home dialysis initiation after January 2021 was statistically significant (*P* = .002). As expected, based on our primary model that included the entire incident dialysis population, the increase in slope of home dialysis use after January 2021 did not differ significantly in ETC and non-ETC markets for Medicare FFS beneficiaries or for those without Medicare FFS coverage.

**Table 3.** Analyses Stratified by Medicare Fee-for-Service Coverage

Metric	Estimate (95% CI)		
	Before January 2021	After January 2021	Difference, after vs before January 2021
**Medicare FFS**			
Utilization of home dialysis, %			
Difference to ETC vs non-ETC^a^	−0.87 (−2.01 to 0.27)	0.10 (−1.29 to 1.50)	0.98 (0.03 to 1.92)
Rate of increase of home dialysis utilization to % per year			
Overall^a^	0.82 (0.70 to 0.94)	1.03 (0.38 to 1.69)	0.22 (−0.45 to 0.88)
Difference to ETC vs non-ETC^b^	−0.21 (−0.37 to 0.06)	0.44 (−0.99 to 1.86)	0.65 (−0.27 to 2.27)
**Other coverage**			
Utilization of home dialysis to %			
Difference to ETC vs non-ETC	−0.74 (−2.1 to 0.62)	0.33 (−1.32 to 1.97)	1.07 (−0.08 to 2.22)
Rate of increase of home dialysis utilization to % per year			
Overall	0.88 (0.74 to 1.02)	2.22 (1.57 to 2.86)	1.34 (0.68 to 2.00)
Difference to ETC vs non-ETC^b^	−0.11 (−0.38 to 0.17)	1.00 (−0.52 to 2.53)	1.11 (−0.42 to 2.64)

Abbreviation: ETC, End-Stage Renal Disease Treatment Choices.

^a^ Estimates are based on the final model with main effects of time, ETC, and pre-post implementation, and 2-way interactions of ETC and pre-post implementation and of time and pre-post implementation. The 2-way interactions of time and ETC assignment and of time and pre-post implementation, and the 3-way interaction of time, ETC assignment, and pre-post implementation were not statistically significant, and thus were removed from the final model.

^b^ Estimates are based on the model and included the 3-way interaction of time, ETC assignment, and pre-post implementation and all 2-way interactions (time and ETC, time and pre-post implementation, ETC and pre-post implementation) to examine whether any changes in use trends after ETC onset differed by ETC assignment.

### Sensitivity Analyses

Results of a sensitivity analysis that excluded patients who initiated dialysis in 2020 were similar to the results of the primary analysis (eTable in Supplement 1). However, when we used September 2020 as the ETC starting point, we found that the increase in rate of increase in home dialysis initiation after September 2020 was significantly greater in ETC markets than in non-ETC markets (difference of 1.32% per year, 95% CI, 0.16%-2.34% per year). The estimates of the effect sizes of ETC implementation after adjustment for patient characteristics were consistent with the unadjusted estimates (eTable in Supplement 1).

## Discussion

In the first 18 months of ETC, initiation of home dialysis increased approximately 1% more in ETC markets than in non-ETC markets. Furthermore, although use of home dialysis among incident dialysis patients has been increasing over the past several years, the rate of increase doubled in the first 18 months of implementation of the ETC payment model. This overall acceleration is notable because only 31.2% of patients received care in markets assigned to ETC participation, and only 33.6% of patients were covered by FFS Medicare as the primary payer. These results suggest that the ETC was associated with increases beyond patients covered by the program; analyses stratified by Medicare FFS coverage not only confirm this but further suggest that the results of ETC were greater among patients without FFS coverage, highlighting the difficulty of increasing home dialysis use in the Medicare FFS population in particular.

The ETC model appears to have had a relatively immediate association with the behavior of health care professionals providing nephrology and dialysis services, highlighting the power of financial incentives. The ESRD Prospective Payment System and other CMS initiatives previously demonstrated the powerful impact CMS payment models can exert when Medicare is the predominant payer, and home dialysis use had been increasing prior to ETC, likely because of some of these initiatives. However, our results suggest that, even when Medicare FFS is not the largest payer, CMS payment changes may be associated with the behavior of health care professionals providing nephrology services. We speculate that clinicians and facilities that provide dialysis in ETC markets may have responded to ETC assignment by implementing changes in practice for all patients rather than only for those with Medicare FFS coverage in the interest of efficiency or equity. Furthermore, the widespread discussion and publication of US government policy to increase home dialysis may have influenced clinicians and facilities providing nephrology and dialysis services in non-ETC markets to also prioritize expansion of home dialysis use.

The apparent initial success of ETC is also remarkable because ETC was implemented with random allocation and mandatory participation to avoid selection bias and ensure generalizeability.^4^ Under a voluntary model, clinicians and facilities providing nephrology and dialysis services who already had higher rates of home dialysis and transplant would have been more likely to volunteer, but mandatory participation and random assignment should ensure that different types of practices and facilities are included in ETC and non-ETC markets and that observed outcomes are the result of implementation of the model itself.

A recent study reported that rates of home dialysis among Medicare FFS beneficiaries were not statistically significantly different in ETC and non-ETC markets in the first 9 months of 2021.^9^ That study relied exclusively on Medicare claims and was thus limited to Medicare FFS beneficiaries aged 66 years or older who initiated dialysis during the first 9 months of 2021; the first part of that interval was characterized by substantial COVID-19–related morbidity among patients undergoing dialysis.^3^ Our results extend follow-up to 18 months and to all patients initiating dialysis, regardless of age or payer. Our analysis suggests that, despite similar rates of initiation of home dialysis in ETC and non-ETC markets after 2021, a change occurred from before 2021 (for Medicare FFS beneficiaries and those without this coverage). Thus, our conclusions differ from those of the aforementioned study.

Although the overall rate of increase in use of home dialysis doubled in the first 18 months of implementation of the ETC payment model compared with before 2021 and the percentage of patients initiating home dialysis increased in ETC markets compared with non-ETC markets, the absolute magnitude of these changes was small. This rate of increase in initiation of home dialysis, if sustained, would lead to rates of initiation of home dialysis comparable to those currently observed in other Western countries (eg, Canada [25.5%]^3^ and Australia [25.2%]^10^) within a decade, whereas it would have taken approximately 2 decades to reach this level had pre-2021 rates of increase continued. Nevertheless, these clinically relevant and statistically significant early increases in home dialysis initiation after ETC implementation are clearly not large enough to achieve ambitious targeted increases in home dialysis use by the end of the payment model in 2027. Reaching the level of home dialysis use observed in these^10,11^ and other countries will likely require more tools than the financial bonuses and penalties that are implemented in ETC. Formalized processes for modality education, rapid reporting of home dialysis use data, streamlined regulatory processes for home dialysis device innovation, and coverage of staff assistance for home dialysis may be just as important to increasing home dialysis use as are adjustments to Medicare reimbursement rates.^11^ Thus, these initial results could be interpreted as meaningful progress but failure to achieve radical change.

### Strengths and Limitations

This study has strengths and limitations. Inclusion of the whole dialysis population in the US and availability of data through the first half of 2022 are strengths. However, limitations related to study design choices deserve comment. We chose the start of ETC implementation as the point of change in our interrupted time series analysis, but ETC assignments were announced in September 2020, and facilities and professionals providing nephrology services may have begun to react in the quarter before this. Sensitivity analysis starting using September 2020 as the start of ETC implementation may support this possibility as there was a statistically significantly larger increase in the rate of increase in home dialysis use after ETC implementation in ETC markets than in non-ETC markets in this analysis. Although the nephrology group practice (and individual nephrologists) are potential sources of correlation among patients, we were not able to cluster at the nephrology clinician level. However, misspecification of the working correlation structure should have little impact on the results of our generalized estimating equation models when the sample size is large, as it is in this study. Our study did not include outcomes among patients undergoing dialysis at home and did not examine transplant waitlisting—another outcome incentivized in the ETC model. Although it will be extremely important to examine outcomes such as the duration of home dialysis therapy, these data will require more time to accrue. Despite these limitations, our study provides an early look at one of the key outcomes of ETC.

## Conclusions

This cohort analysis noted that use of home dialysis increased more among patients in ETC markets compared with non-ETC markets in 2021 and early 2022, suggesting immediate success. Immediate increases in home dialysis use suggest that professionals providing nephrology services responded to financial bonuses by increasing use of home dialysis, but it will be important to monitor whether this increase continues when the incentive structure changes to performance payment adjustments.

## Notes

Supplement 1. **eTable.** Results of Sensitivity Analyses **eFigure.** Monthly Proportion of Incident Dialysis Patients Receiving Home Dialysis by Medicare Fee for Service (FFS) Coverage

Supplement 2.
Data Sharing Statement
