# Supplementary material for: Association of the End-Stage Renal Disease Treatment Choices Payment Model With Home Dialysis Use at Kidney Failure Onset From 2016 to 2022
Source: JAMA Netw Open. 2023 Feb 27;6(2):e230806. doi: 10.1001/jamanetworkopen.2023.0806 (PMC9972188; doi:10.1001/jamanetworkopen.2023.0806)
Supplement: Supplement 1. — eTable. Results of Sensitivity Analyses eFigure. Monthly Proportion of Incident Dialysis Patients Receiving Home Dialysis by Medicare Fee for Service (FFS) Coverage [file jamanetwopen-e230806-s001.pdf]

Supplementary Online Content

Johansen KL, Li S, Liu J, et al. Association of the End-Stage Renal Disease Treatment Choices payment model with home dialysis use at kidney failure onset from 2016 to 2022. *JAMA Netw Open*. 2023;6(2):e230806. doi:10.1001/jamanetworkopen.2023.0806

eTable. Results of Sensitivity Analyses

eFigure. Monthly Proportion of Incident Dialysis Patients Receiving Home Dialysis by Medicare Fee for Service (FFS) Coverage

This supplementary material has been provided by the authors to give readers additional information about their work.

© 2023 Johansen KL et al. *JAMA Network Open.*

eTable. Results of sensitivity analyses

		**Before January 2021**	**After January 2021**	**Difference, after vs. before January 2021**
**Type of sensitivity analyses**	**Metric**	Estimate (95% CI)	Estimate (95% CI)	Estimate (95% CI)
**Unadjusted, excluding 2020 data**	**Use of home dialysis, %**			
	Difference, ETC vs. non-ETC[a]	-0.80 (-2.00, 0.40)	0.17 (-1.23, 1.58)	0.97 (0.02, 1.92)
	**Rate of increase of home dialysis use, % per year**			
	Overall[a]	0.76 (0.63, 0.89)	1.67 (1.15, 2.18)	0.90 (0.38, 1.43)
**Starting ECT period in September 2020, unadjusted**	**Use of home dialysis, %**			
	Difference, ETC vs. non-ETC[b]	–	–	–
	**Rate of increase of home dialysis use, % per year**			
	Overall[b]	–	–	–
	Difference, ETC vs. non-ETC[c]	-0.15 (-0.37, 0.07)	1.17 (0.02, 2.31)	1.32 (0.16, 2.48)
**Adjusted for patient characteristics^**	**Use of home dialysis, %**			
	Difference, ETC vs. non-ETC[a]	-0.10 (-1.20, 1.00)	1.02 (-0.20, 2.24)	1.11 (0.27, 1.96)
	**Rate of increase of home dialysis use, % per year**			
	Overall[a]	0.87 (0.77, 0.98)	1.68 (1.19, 2.18)	0.81 (0.30, 1.31)

[a]Estimates are based on the final model with main effects of time, ETC, and pre-/post-implementation, and two-way interactions of time and pre-/post-implementation and of ETC and pre-/post-implementation. The two-way interaction of time and ETC assignment and the three-way interaction of time, ETC assignment, and pre-/post-implementation were not statistically significant, thus removed from the final model.

[b]Estimates are not provided in the presence of significant three-way interaction of time, ETC assignment, and pre-/post-implementation.

[c]Estimates are based on the model included the three-way interaction of time, ETC assignment, and pre-/post-implementation and all two-way

© 2023 Johansen KL et al. *JAMA Network Open.*

interactions (time and ETC, time and pre/post, ETC and pre/post) to examine whether any changes in trends in home dialysis use after ETC onset differed by ETC assignment.
^Patient characteristics in the model included age groups, race/ethnicity, rural/urban status, and Medicare FFS enrollment status.

© 2023 Johansen KL et al. *JAMA Network Open.*

eFigure. Monthly proportion of incident dialysis patients receiving home dialysis by Medicare fee for service (FFS) coverage

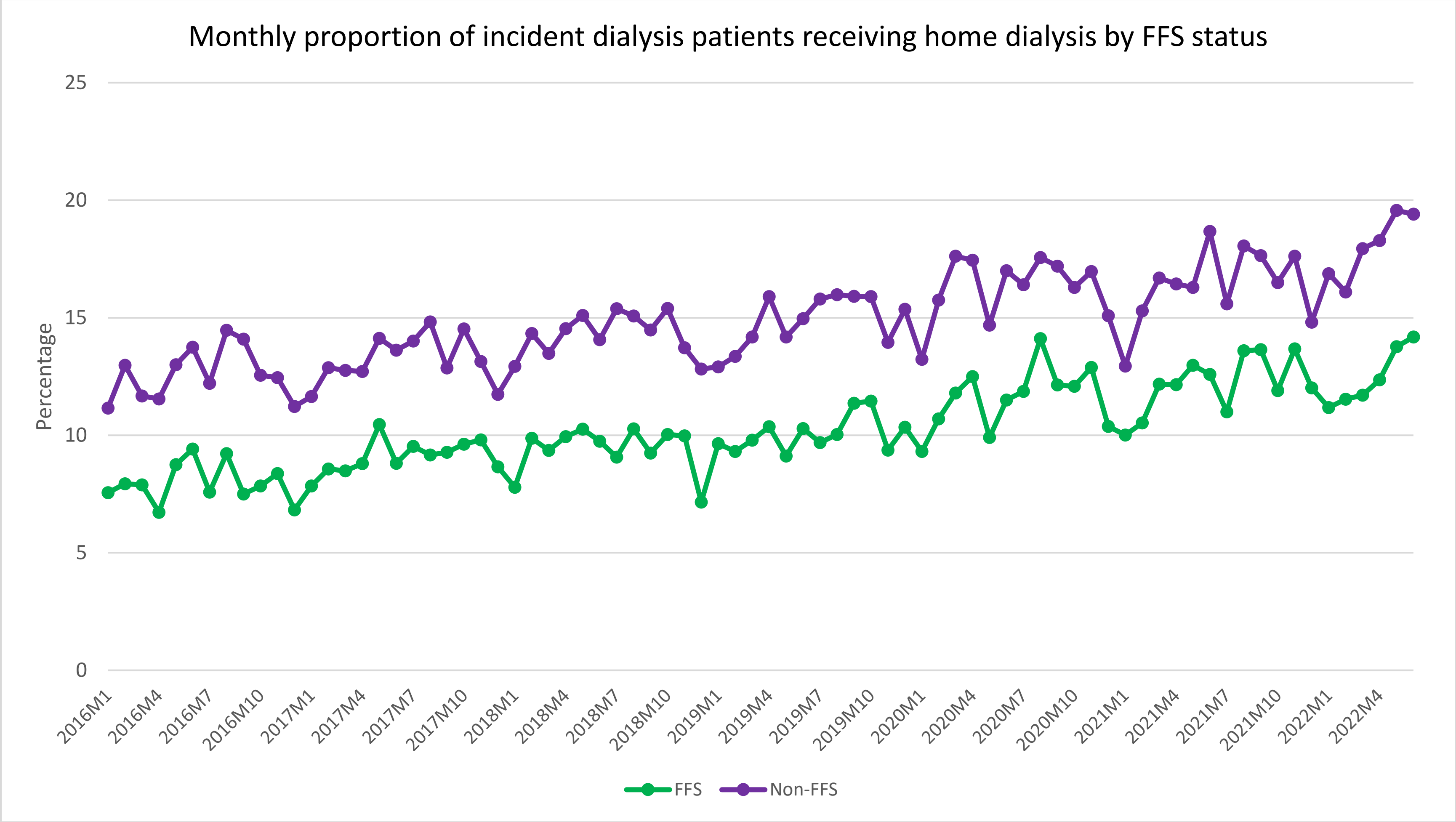

© 2023 Johansen KL et al. *JAMA Network Open.*
