# Supplementary material for: Association of the End-Stage Renal Disease Treatment Choices Payment Model With Home Dialysis Use at Kidney Failure Onset From 2016 to 2022
Source: JAMA Netw Open. 2023 Feb 27;6(2):e230806. doi: 10.1001/jamanetworkopen.2023.0806 (PMC9972188; doi:10.1001/jamanetworkopen.2023.0806)
Supplement: Supplement 2. — Data Sharing Statement [file jamanetwopen-e230806-s002.pdf]

Data Sharing Statement

Johansen. Association of the End-Stage Renal Disease Treatment Choices Payment Model With Home Dialysis Use at Kidney Failure Onset From 2016 to 2022. *JAMA Netw Open*. Published February 27, 2023. doi:10.1001/jamanetworkopen.2023.0806

Data
Data available: No

Additional Information
Explanation for why data not available: The data used in the study were supplied by the Centers for Medicare and Medicaid Services and cannot be rereleased to others. However, investigators could request the data directly from CMS.
